# The Vitamin D Receptor Inhibits the Respiratory Chain, Contributing to the Metabolic Switch that Is Essential for Cancer Cell Proliferation

**DOI:** 10.1371/journal.pone.0115816

**Published:** 2014-12-29

**Authors:** Marco Consiglio, Michele Destefanis, Deborah Morena, Valentina Foglizzo, Mattia Forneris, Gianpiero Pescarmona, Francesca Silvagno

**Affiliations:** 1 Department of Oncology, University of Torino, Torino, Italy; 2 Center for Experimental Research and Medical Studies, S. Giovanni Battista Hospital, Torino, Italy; 3 Department of Molecular Biotechnology and Health Sciences, Molecular Biotechnology Center, University of Torino, Italy; Nihon University School of Medicine, Japan

## Abstract

We recently described the mitochondrial localization and import of the vitamin D receptor (VDR) in actively proliferating HaCaT cells for the first time, but its role in the organelle remains unknown. Many metabolic intermediates that support cell growth are provided by the mitochondria; consequently, the identification of proteins that regulate mitochondrial metabolic pathways is of great interest, and we sought to understand whether VDR may modulate these pathways. We genetically silenced VDR in HaCaT cells and studied the effects on cell growth, mitochondrial metabolism and biosynthetic pathways. VDR knockdown resulted in robust growth inhibition, with accumulation in the G0G1 phase of the cell cycle and decreased accumulation in the M phase. The effects of VDR silencing on proliferation were confirmed in several human cancer cell lines. Decreased VDR expression was consistently observed in two different models of cell differentiation. The impairment of silenced HaCaT cell growth was accompanied by sharp increases in the mitochondrial membrane potential, which sensitized the cells to oxidative stress. We found that transcription of the subunits II and IV of cytochrome c oxidase was significantly increased upon VDR silencing. Accordingly, treatment of HaCaT cells with vitamin D downregulated both subunits, suggesting that VDR may inhibit the respiratory chain and redirect TCA intermediates toward biosynthesis, thus contributing to the metabolic switch that is typical of cancer cells. In order to explore this hypothesis, we examined various acetyl-CoA-dependent biosynthetic pathways, such as the mevalonate pathway (measured as cholesterol biosynthesis and prenylation of small GTPases), and histone acetylation levels; all of these pathways were inhibited by VDR silencing. These data provide evidence of the role of VDR as a gatekeeper of mitochondrial respiratory chain activity and a facilitator of the diversion of acetyl-CoA from the energy-producing TCA cycle toward biosynthetic pathways that are essential for cellular proliferation.

## Introduction

The vitamin D receptor (VDR), along with the other members of the steroid hormone receptor family, has generally been described as a classical ligand-modulated transcription factor. The differentiating effects of the VDR are triggered by ligand-induced nuclear translocation and binding to vitamin D responsive element (VDRE) sites on regulated genes, in association with heterodimerization partners, coactivators and corepressors. Differences in corepressor binding and DNA methylation reflect the profound variability of VDR antiproliferative responses in different cell models [1]. Resistance to the nuclear effects of vitamin D has been reported in several models of cancer, including prostate, breast and bladder cancers, in which increased corepressor expression and localization has been deemed to be responsible for the insensitivity to the hormone [2]–[4].

Steroid receptors also possess a nongenomic modality of action, particularly at plasma membrane or mitochondrial sites, and the VDR is no exception. In fact, the rapid, nongenomic effects of vitamin D appear to be mediated by the VDR [5]–[7]. Many steroid receptors and nuclear transcription factors enter the mitochondrial compartment, where they either exert transcriptional regulation of mitochondrial DNA or control mitochondrial biogenesis and metabolism [8]–[11]. We recently described the mitochondrial localization of the VDR in a human proliferating keratinocyte cell line (HaCaT) for the first time and demonstrated that mitochondrial import of the receptor is mediated by the permeability transition pore complex [12]. However, the function of the VDR in this organelle remains to be elucidated. Mitochondria are multifunctional organelles and mitochondrial activity is important for cellular proliferation and physiology. For example, the mitochondria play essential roles in cellular energy (ATP) production via the tricarboxylic acid (TCA) cycle coupled to oxidative phosphorylation (OXPHOS), as well as during apoptosis via reactive oxygen species (ROS) generation and cytochrome c release. Several studies have indicated that mitochondrial dysfunction contributes to the development and progression of various human diseases, including cancer [13]. A hallmark of tumor cells is altered metabolism supporting rapid cellular proliferation [14]. Many metabolic intermediates that support cell growth are provided by the mitochondria [15]; consequently, the identification of proteins that regulate mitochondrial metabolic pathways is of great interest, and we sought to understand whether the VDR may modulate these pathways.

In the present study, using our previously described model (the proliferating HaCaT cell line), we genetically silenced the receptor and examined the effects on cell growth, mitochondrial metabolism and biosynthetic pathways. The collected data provide evidence of a novel role of the VDR as a negative regulator of respiratory chain activity, and we highlight the repercussions for cellular anabolism and growth produced by the VDR on mitochondrial respiration. Based on our observations, we conclude that the VDR, by restraining mitochondrial respiratory activity, allows the cell to spare metabolic intermediates, which may be diverted from oxidative metabolism toward a biosynthetic fate, supporting cell growth. We validated the general role of the VDR as an enhancer of cellular proliferation extending our observations to several human cancer cell lines.

## Results

### VDR silencing in HaCaT cells hampers cellular proliferation

**Figure 1:**
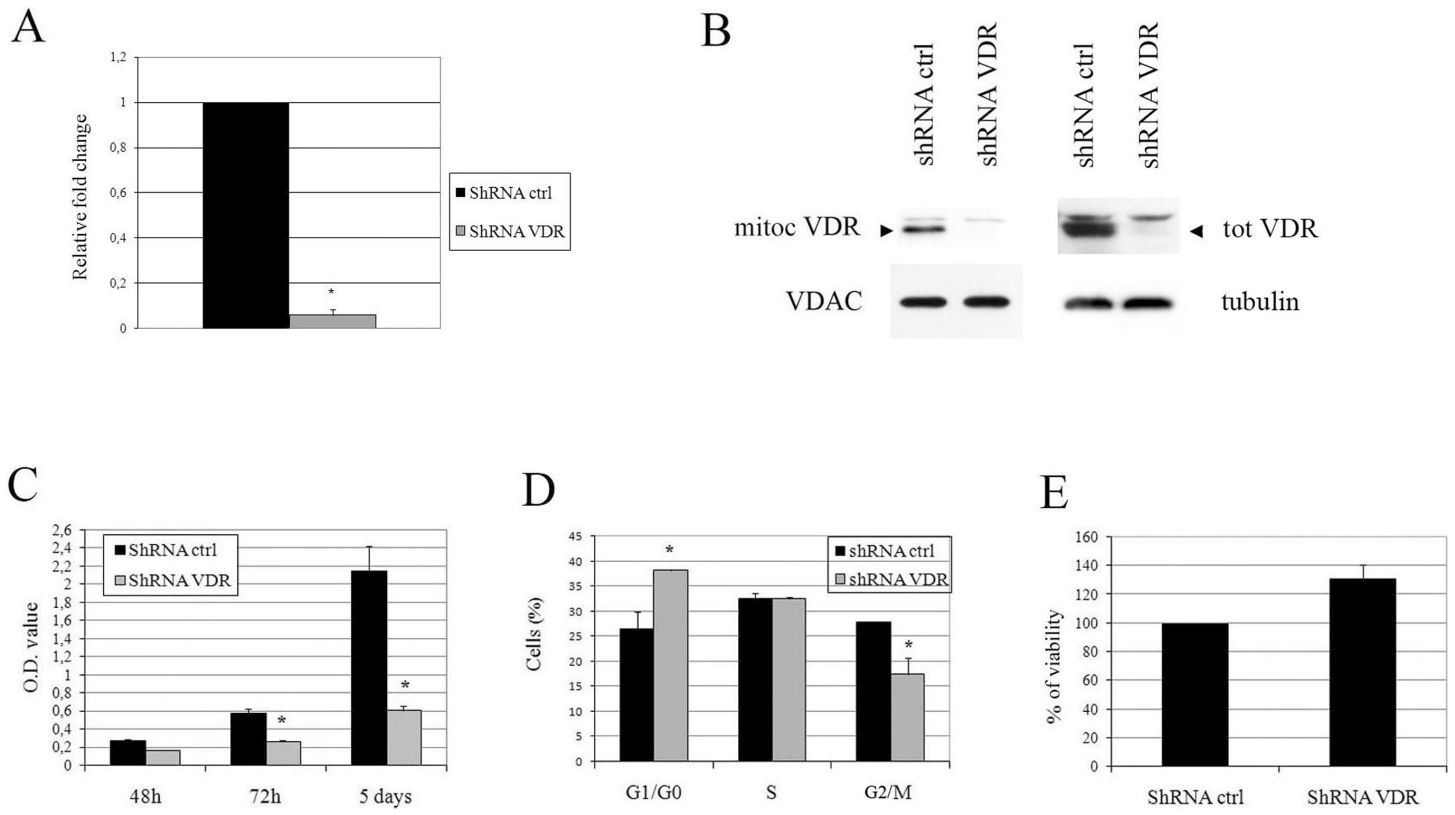
shRNA-mediated VDR knock down in HaCaT cells nearly abolishes VDR expression and drastically reduces cell growth. Subconfluent HaCaT cells were infected with lentiviral VDR shRNA 3 and shRNA control particles. Seven days after infection and puromycin selection, VDR expression was evaluated in the cellular extracts. (**A**) mRNA expression levels were quantified using real-time analysis of VDR transcripts, and the values are expressed as fold changes in the silenced cells compared to the controls. (**B**) VDR expression in the mitochondrial fractions (left panels) and total lysates (right panels) from shRNA-transfected control and shRNA-transfected VDR cells was analyzed using western blotting. Tubulin detected in total extracts and VDAC levels in mitochondrial fractions were used as internal controls for protein loading. The effects of VDR silencing on proliferation were evaluated using a crystal violet assay in cells that had been stained at various times after seeding (**C**), as well as using cell cycle analysis (**D**) in cells that were harvested at day 7 post-infection. (**E**) Cell viability was evaluated using the MTT assay at day 7 post-infection and the values are expressed as the percentage of absorbance of the shRNA control. The data are expressed as the means ±SD of three independent experiments. * P<0.05 compared to the control.

Because we previously characterized VDR mitochondrial import in HaCaT cells, we used this model to investigate VDR function using genetic silencing. The abatement of VDR levels upon lentiviral delivery of shRNA that had been raised against the human VDR was remarkable, as demonstrated by mRNA and protein analyses in fig. 1A and 1B, and triggered potent growth arrest in HaCaT cells, as evaluated using a proliferation assay, which highlighted an evident decrease in the growth rate in VDR-silenced cells compared to control cells (fig. 1C). Cell growth arrest was characterized by the accumulation of the VDR knock down cell population in the G0/G1 phase of the cell cycle and a decrease in the M phase cell population (fig. 1D). No signs of apoptotic or suffering cells were evident in the silenced cells, as demonstrated by cell cycle analysis, which did not reveal a sub-G0 peak, and the MTT toxicity assay, which revealed identical viability in the control and silenced cells (fig. 1E).

The differentiating and antiproliferative action of vitamin D in vitro has been previously described in literature. Such effects are mediated by transcriptional control, which is preceded by nuclear translocation and does not occur in vitamin D-stimulated HaCaT cells [12]. HaCaT cells appear to be resistant to the nuclear antiproliferative effects of vitamin D, and accordingly, we found that vitamin D treatment did not alter the growth rate of HaCaT cells (as shown in S1 fig.). Thus, HaCaT cells represent a model of resistance to the differentiating properties of vitamin D, and there is not any incongruity between the nuclear antiproliferative role of vitamin D described in literature and the proliferative effects exerted by VDR in our cell model. The results of our silencing experiments show that VDR in HaCaT cells enhances cell growth.

### Mitochondrial localization of the VDR is a common feature of human cancer cell lines, and receptor silencing inhibits cellular proliferation

**Figure 2:**
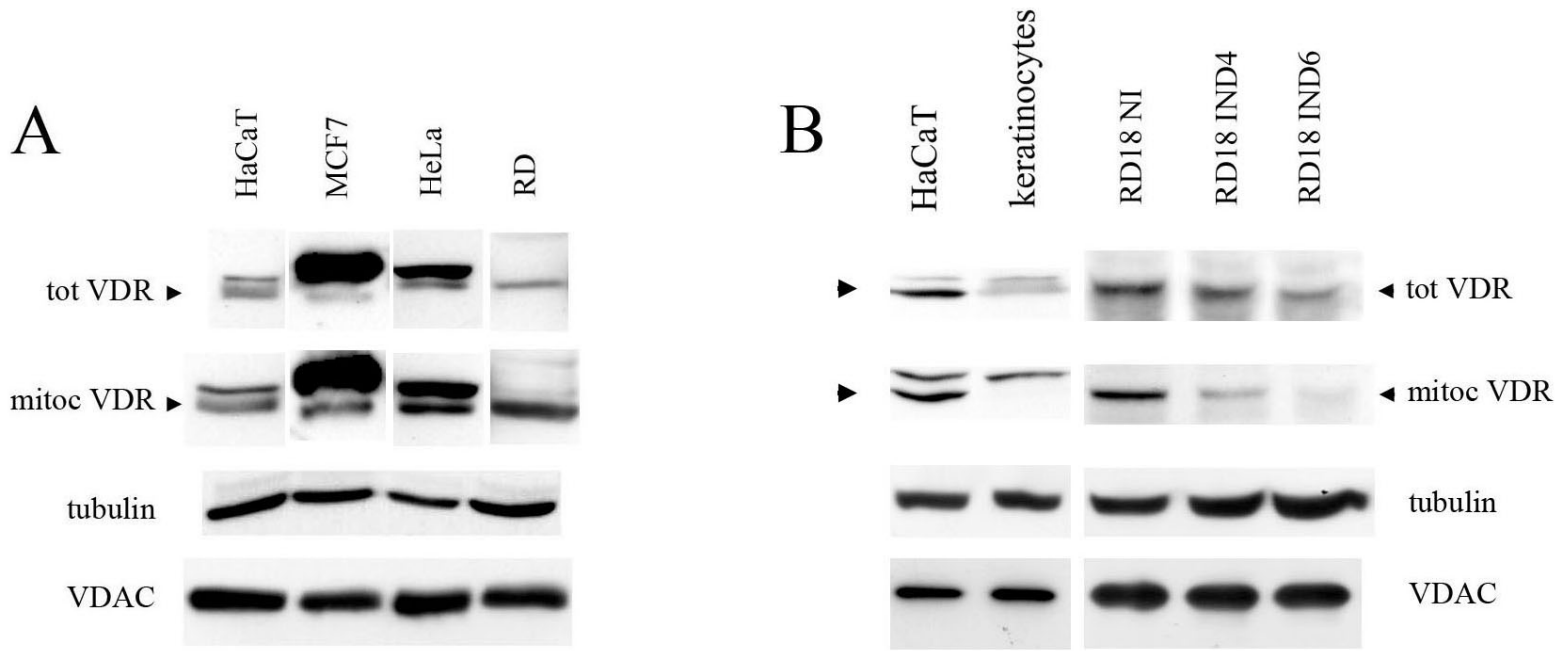
Proliferating human cells express mitochondrial VDR, whereas differentiated cells display reduced levels of receptor expression. (**A**) VDR expression was analyzed in a panel of several human cell lines using western blotting in total lysates (tot VDR) and mitochondrial extracts (mitoc VDR). For RD and MCF7 cells, VDR detection required a longer duration of exposure to ECL. (**B**) Two models of cellular differentiation were used to assess VDR levels in the total lysates and mitochondrial fractions: Human proliferating HaCaT cells vs. human primary differentiated keratinocytes and differentiation-inducible RD18 cells carrying a doxycycline-inducible miR-206-expressing lentiviral vector in the absence (uninduced: NI) or presence of doxycycline for four (induced: IND4) and six days (induced: IND6). Tubulin detected in total extracts and VDAC levels in mitochondrial fractions were used as internal controls for protein loading. The blots are representative of a set of three independent experiments.

In order to assess whether the mitochondria may be considered to be a regular target of the VDR, we evaluated its expression in a panel of human cancer cell lines and the aforementioned HaCaT cells (fig. 2A). In every cell line that tested positive for VDR expression, the mitochondrial extracts were analyzed via western blotting and revealed the presence of the receptor. We hypothesized that if the VDR was a hallmark of proliferating cells and had the potential to facilitate cell growth, as suggested by the phenotype observed in silenced HaCaT cells, then it could easily be spared, or even removed, in a differentiated state. Thus, we evaluated VDR expression levels in two models, allowing for the examination of human proliferating cells and their differentiated counterparts: We compared HaCaT proliferating keratinocyte cells to fully differentiated primary keratinocytes and rhabdomyosarcoma RD18 cells that had been induced to differentiate by the conditional expression of miR-206 [16]. Mitochondrial extracts of both differentiated cell populations revealed decreased receptor expression as compared to the levels observed in proliferating cells (fig. 2B).

**Figure 3:**
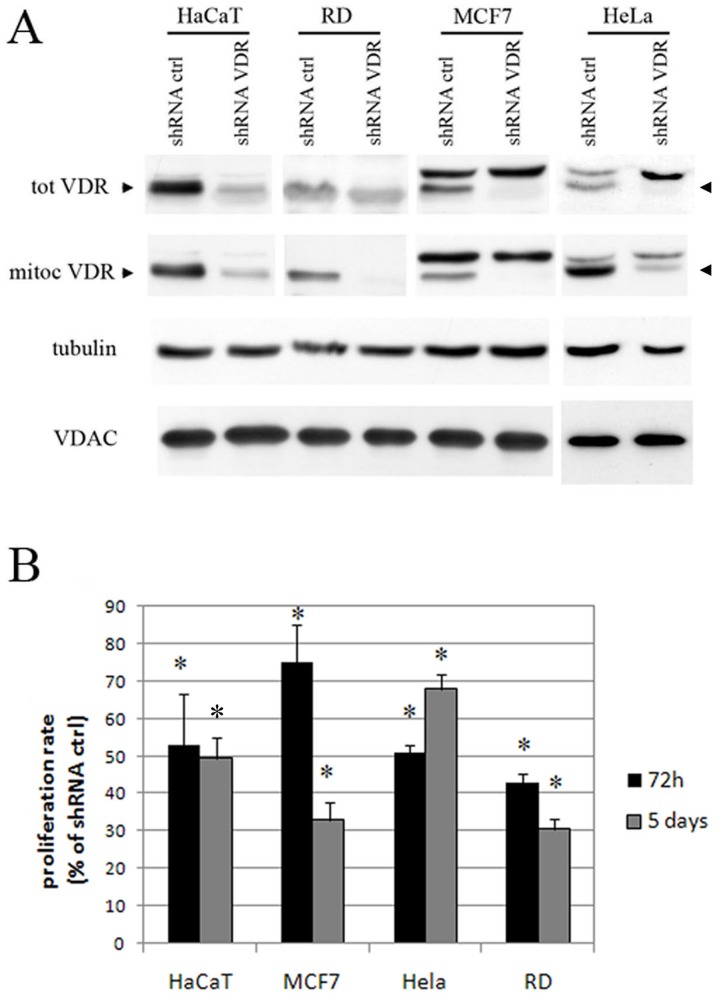
VDR silencing inhibits the proliferation of several human cancer cell lines. (**A**) The cells were infected with lentiviral VDR shRNA 3 or shRNA control and the silencing efficacy was examined in both the total and mitochondrial extracts using western blotting. Tubulin detected in total extracts and VDAC levels in mitochondrial fractions were used as internal controls for protein loading. (**B**) Both the silenced and control cells were subjected to proliferation assays seven days after infection and selection. The cells were stained at 72 hours or five days after seeding, and the values for the silenced cells are expressed as the percentage of their respective controls. The data are expressed as the means ±SD of three independent experiments. * P<0.05 compared to the control.

Having detected the widespread mitochondrial expression of the VDR, we sought to confirm that VDR ablation compromises cellular proliferation; thus, we abolished VDR expression in all of the cancer cell lines using genetic silencing. VDR expression was silenced in all of the VDR-positive cell lines, as well as the HaCaT cells, and receptor expression was once again strongly reduced, as evaluated both in the total extracts and the mitochondrial fractions (fig. 3A). The observations made in HaCaT cells were strongly supported by the results of these knockdown experiments because the proliferation of all of the cells was markedly inhibited by VDR silencing. In fact, we were able to demonstrate a general reduction in the proliferation rate of all of the silenced cells, which was similar to that observed in the HaCaT cells, when their growth was evaluated between 72 h and 5 days as compared to that of wild type cells (fig. 3B). The silenced phenotype was confirmed using a different shRNA for the VDR (ShRNA 4, as described in the Methods section), which was as efficient in silencing the VDR as ShRNA 3 and also decreased cell growth (S2 fig.). Moreover, when we delivered an shRNA that was ineffective in terms of VDR silencing (ShRNA 2, as described in the Methods section) the proliferation of the HaCaT cells was not constrained (S2 fig.).

### VDR silencing enhances mitochondrial respiration

**Figure 4:**
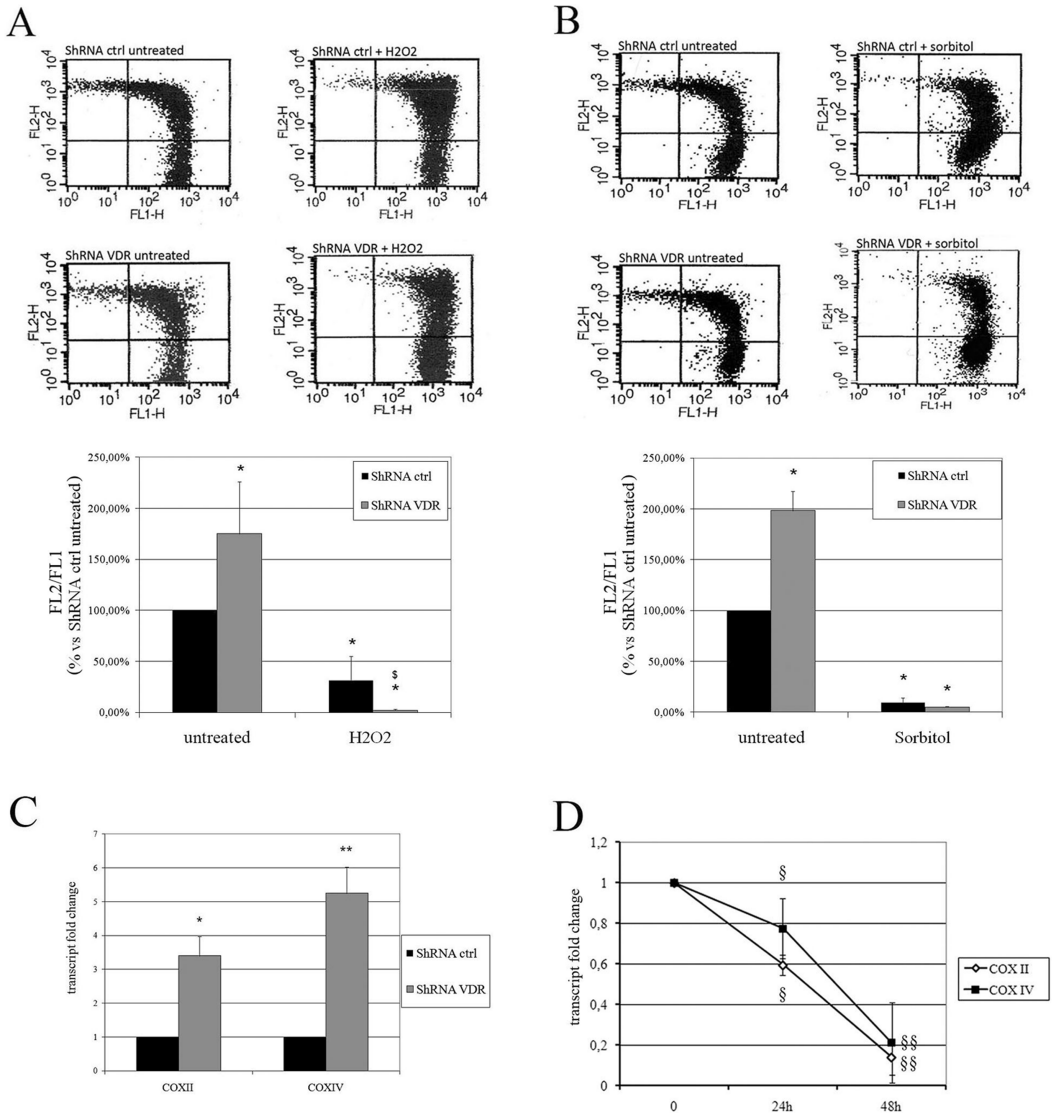
Effects of VDR silencing on mitochondrial activity. HaCaT cells were infected with shRNA control or VDR shRNA 3 and the mitochondrial membrane potential was examined using JC-1 cytofluorimetric evaluation, in the presence or absence of two different stressors: (**A**) Control and silenced cells were treated with either 10 mM H_2_O_2_ or (**B**) 0.5 M sorbitol. In both figures, a representative image from the cytofluorimetric analysis is shown in the top panel, whereas the results from three separate experiments are plotted in the graph in the lower panel. The FL-2/FL-1 ratio was calculated and the values are expressed as a percentage of the untreated shRNA control. * P<0.05 compared to the untreated shRNA control, **^$^** P<0.05 compared to the treated shRNA control. (**C**) Real time analysis of COX II (COX II) and IV (COX IV) subunit transcript expression in control and silenced cells. Fold changes are plotted on the graphs as the means ±SD of three independent experiments. * P<0.05 and ** P<0.01 compared to the shRNA control. (**D**) HaCaT cells were grown in the presence or absence of 10 nM vitamin D and COX II and COX IV transcript expression were evaluated using real-time analysis after 24 and 48 hours of treatment. The values plotted on the graphs represent the fold change in transcript expression in treated versus untreated cells and are displayed as the means ±SD of three independent experiments. **^§^** P<0.05 and **^§§^** P<0.01 compared to the untreated cells.

Other steroid receptors have been previously indicated to regulate mitochondrial transcription and activity [17]–[19], prompting us to investigate whether the VDR, which is localized in the mitochondrial compartment (similar to its family analogs), controls mitochondrial metabolism. First, we evaluated the mitochondrial respiratory activity of HaCaT cells by measuring variations in membrane potential via JC-1 analysis using cytofluorimetry. As shown in fig. 4A, VDR silencing strongly increased the mitochondrial membrane potential, and when we subjected these cells to oxidative stress (H_2_O_2_), their potential was impaired to a larger extent than that of wild type cells subjected to the same treatment (fig. 4A). However, when the cells were exposed to a different, non-oxidative stress (e.g., sorbitol-induced osmotic stress), the mitochondrial potential of both wild type and knock down cells decreased to the same extent (fig. 4B). Based on these observations, we concluded that the VDR inhibited the mitochondrial membrane potential and likely restrained ROS production, protecting the cell from additional oxidative stress. On the contrary, VDR loss increased the respiratory potential, but rendered cells more prone to an oxidant-driven potential collapse. This possibility was supported by the significantly lower glutathione (GSH) consumption in wild type cells, as revealed by the higher levels of the antioxidant molecule that were measured in wild type cells compared to silenced cells (S3 fig.).

Mitochondrial potential is sustained by the proton gradient that is created by respiratory chain activity; therefore, we decided to examine the expression of two subunits of complex IV: Cytochrome c oxidase (COX) subunits II and IV, whose transcripts are of mitochondrial (the former) and nuclear (the latter) origin. Both nuclear- and mitochondrially encoded proteins are required for the formation of active respiratory complexes. Mitochondrial RNAs are transcribed as long, polycistronic precursor transcripts that are later processed to release individual rRNAs and mRNAs. Therefore, we considered COX II to be a marker of mitochondrial transcription activity and COX IV to be a marker of the nuclear contribution to respiratory chain modulation.

Increased expression of both subunits in silenced cells compared to control cells was observed using real-time PCR (fig. 4C). In order to confirm that the VDR negatively affected COX transcription, we treated wild type HaCaT cells with vitamin D and observed that the levels of all of the transcripts were decreased (fig. 4D). Given the fact that the transcription of subunit IV is nuclear, whereas that of subunit II is encoded by mitochondrial DNA (mtDNA), we concluded that vitamin D transcriptional control is exerted at both levels, which is not surprising given the fact that nuclear and mitochondrial transcription of respiratory chain proteins is finely tuned [20].

Because the modulation of mitochondrial transcription by the VDR has not been previously described, we considered the possibility of direct binding of the receptor to mtDNA. *In silico* analysis was conducted with the aim of screening mtDNA to identify vitamin D responsive element (VDRE) sites. We used a VDRE sequence represented by a collection of positional weight matrices (as described in the Methods section and displayed in S1B table) to compute the affinity of the VDR for the mtDNA sequence. Only two VDRE sites were found to have high affinity cutoffs (89% and 82% of the maximum score) and both were located in the displacement loop (D-loop), a non-coding and regulatory region. We also identified a total of 40 VDRE sites with low affinity scores (>60% of the maximum) clustered in a few regions (see table 1 for a description of the higher affinity VDRE sites and S1A table for the complete list). In some cases, these VDRE sites are formed by multiple repeats, indicating a significant presence of binding sites in these regions. Of note, the vast majority of VDRE sites were found on the reverse strand, which is likely due to the skewness of mtDNA nucleotide distribution. In addition, low affinity sites were enriched on hypervariable segment 1 (p = 0.01425). The high affinity of the identified VDRE sites allows for the possibility of VDR-mediated direct transcriptional control of mtDNA. Further studies are planned to investigate the characteristics of this binding.

**Table 1:** Highest affinity VDRE sites in the mtDNA sequence, as detected using *in silico* analysis.

MATRIX	START SITE	SCORE	STRAND	MATRIX	START SITE	SCORE	MATRIX	START SITE	SCORE
DR4	1220	76.88%	-	DR3	1230	72.21%			
DR4	1481	63.75%	-	DR3	1491	72.21%			
DR4	2249	72.51%	-						
EV7	3580	60.92%	-	EV6	3581	75.10%	EV8	3585	73.65%
EV6	4050	73.09%	-						
DR4	5323	76.88%	-	DR3	5323	78.70%			
EV9	5613	72.23%	-	DR4	5618	61.97%			
DR3	5703	70.13%	-	DR4	5712	68.41%	EV8	5712	63.82%
DR4	9637	78.93%	-						
EV9	11142	70.03%	-						
DR4	13292	70.46%	-						
DR4	13444	78.93%	-						
EV7	14050	69.40%	-	EV6	14051	71.08%			
EV7	14390	73.63%	-						
EV9	15384	70.28%	-						
**EV6**	**16056**	**89.96%**	-	**DR4**	**16068**	**70.46%**			
**EV7**	**16205**	**73.63%**	-						
**EV8**	**16225**	**82.30%**	-						

MATRIX: One of the possible VDRE sites matching the mtDNA sequence (the sequence and matrix are described in S1 table). START SITE: The start site of the sequence referred to in the UCSC database (as described in the Methods section). SCORE: The affinity score is reported as a percentage of the maximum score for each matrix. STRAND: The strand of the sequence. For overlapping sequences, more than one matrix, start site and score are reported. Sites in the D-loop are highlighted in bold text.

The increased membrane potential that was observed in the silenced cells, combined with the induced expression of COX transcripts, demonstrated that the respiratory chain was potentiated in silenced HaCaT cells.

Taken together, our results indicate that the VDR plays a role in the negative modulation of respiratory chain expression and respiratory activity, which both moderates membrane potential and is protective against oxidative stress.

### VDR silencing minimizes acetyl-CoA utilization in biosynthetic pathways

In an effort to link the reduced cell growth and the increased mitochondrial potential that was observed in VDR knock down cells, we hypothesized that intense activity of the respiratory chain stimulates the TCA cycle in silenced cells. In quiescent cells, the fundamental function of the mitochondria is to produce ATP via a TCA cycle-feeding electron transport chain and oxidative phosphorylation in order to supply energy for a variety of cellular functions; in contrast, cancer cells are not heavily dependent upon OXPHOS for their energy demands and re-direct TCA cycle intermediates to preserve their biosynthetic function. Thus, in proliferating cells, mitochondrial pathways are rewired to support proliferation.

**Figure 5:**
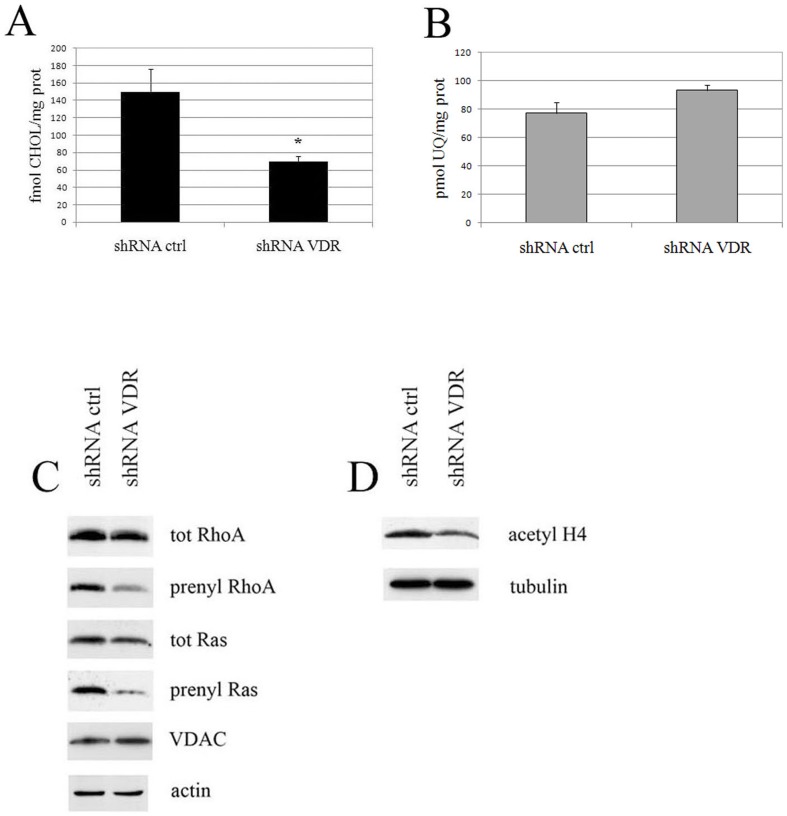
VDR knock down cells display an impaired acetyl-coA-dependent biosynthetic rate. HaCaT cells were infected with shRNA control or VDR shRNA 3 and biosynthetic pathways were examined seven days post-infection. The mevalonate pathway was evaluated as the *de novo* synthesis of cholesterol (**A**) and ubiquinone (**B**). The values represent the means ±SD of three independent experiments. (**C**) Isoprenoid units produced by the same pathway were analyzed as prenyl moiety incorporation in the small GTPases RhoA and Ras. Control and silenced cells were harvested and the lysates were subjected to TX-114 phase-extraction in order to separate the prenylated forms. Total and prenylated proteins were analyzed using western blotting. VDAC and actin expression demonstrated equivalent protein loading of the hydrophobic phase and total extracts, respectively. (**D**) Histone acetylation levels were evaluated by western blotting analysis using an anti-acetyl H4 antibody and tubulin as a loading control. The blots are representative of a set of three independent experiments.

Our data suggested that the VDR, reducing the metabolic demands of the respiratory chain, may reprogram the TCA cycle and its intermediates may be used in biosynthetic processes. In order to test this hypothesis, we analyzed the biosynthetic pathways dependent on acetyl-CoA diverted from mitochondrial catabolism. First, we considered the products of mevalonate cascade, namely cholesterol, ubiquinone and isoprenic units essential for post-translational modifications of proteins. Cholesterol and ubiquinone *de novo* synthesis was measured in HaCaT cells. Control and VDR knock down cells were labeled with [^3^H]acetate and the lipid content of the cells was assayed using TLC. VDR silencing decreased the *de novo* synthesis of cholesterol (fig. 5A), whereas the ubiquinone biosynthetic rate was unaffected by silencing (fig. 5B). The synthesis of isoprenic units was measured as the prenylation status of two small GTPases: RhoA and Ras. VDR silencing resulted in decreased prenylation of both proteins, whereas their overall expression remained unchanged (fig. 5C).

Finally, the availability of acetyl units for biosynthetic purposes was evaluated as histone acetylation and VDR silencing also decreased this process, which was measured as histone H4 acetylation (fig. 5D).

Collectively, these observations indicate that upon VDR silencing, the increased respiratory chain activity oxidizes metabolic intermediates, preventing their utilization in biosynthetic pathways. We demonstrated the exemplary diversion of acetyl-CoA, the incorporation of which is reduced during cholesterol biosynthesis, prenylation events and histone remodeling.

## Discussion

Our previous work in HaCaT cells demonstrated VDR mitochondrial localization and the mechanism of import. In the present study, we identified a strikingly important new role for the receptor in organelle function and cellular metabolism because we demonstrated that VDR activity deeply impacts proliferation.

Mitochondrial functions have previously been described for other steroid receptors, several of which stimulate mitochondrial respiration and are therefore considered to be either differentiating hormone receptors (i.e., the thyroid receptor, estrogen receptor beta and the androgen receptor) or energy expenditure enhancers and providers (i.e., the glucocorticoid receptor) [17], [21], [22]. Hormonal stimulus affects the transcription of mitochondrially encoded OXPHOS both indirectly, by inducing nuclear signals (such as mitochondrial transcription factors, which positively regulate transcription of the mitochondrial genome), and directly, by localizing in the organelle and interacting with response elements in mitochondrial DNA [23]. To date, VDR function in the mitochondria remains uncharacterized.

In the present study, we demonstrated that the VDR promotes proliferation, as its silencing strongly affects the growth rate of HaCaT and other cancer cell lines expressing a mitochondrial VDR. Starting with the previously characterized HaCaT cells and extending our analysis to other cellular models, we found that mitochondrial localization of the receptor is a widespread characteristic of proliferating cells, and the association of the VDR with proliferation was reinforced by the results of our analysis of differentiated cells. We observed decreased mitochondrial VDR levels in two different models of differentiated cells (primary cultures of keratinocytes represent physiological differentiation, whereas miR-206-induced RD18 cells represent an miRNA-driven differentiated state). In our opinion, this is an interesting observation that warrants further investigation of the metabolic impact and molecular mechanisms governing VDR downregulation in quiescent cells.

Previous literature described the differentiating properties of vitamin D, traditionally mediated by nuclear effects of VDR on transcription. However cancer cells are often resistant to the antiproliferative and differentiating properties of vitamin D, as a result of the increased association of the VDR with corepressors on chromatine [24]. This has been reported for skin cancer, among the others [25]. The human proliferating keratinocyte cell line HaCaT does not respond to the antiproliferative action of vitamin D (S1 fig.), and we previously demonstrated that nuclear translocation of the VDR, which is a prerequisite for transcriptional activity, is not induced upon ligand stimulation [12], thus indicating ineffective, or feeble, nuclear VDR signaling in these cells. Therefore, HaCaT cells represent a good model that can be used to examine the mitochondrial effects of VDR activity in a background were the differentiating properties of vitamin D have been lost.

Genetic silencing of the VDR in HaCaT cells produced two effects that were linked: reduced proliferation that corresponded to an increase in respiratory chain expression and the mitochondrial membrane potential. We had evidence that the VDR balances electron chain activity, resulting in dual advantages for the cell: protection from oxidative stress and support for proliferation through readily available biosynthetic intermediates. The former conclusion was reached when we observed the increased vulnerability of silenced cells to a strong oxidative insult, accompanied by a decreased intracellular GSH pool. The latter interpretation, the other major finding of our work, was suggested by the experiments aimed at evaluating the biosynthetic capacity of silenced HaCaT cells. We analyzed different biosynthetic pathways that rely on acetyl-CoA of mitochondrial origin. Acetyl-CoA is a vital building block for the endogenous biosynthesis of fatty acids and cholesterol and is involved in isoprenoid-based protein modifications; acetyl-CoA is also required for acetylation reactions that modify proteins, such as histone acetylation. We found that VDR knock down cells displayed a curtailed biosynthetic rate of the mevalonate pathway, measured as the reduced production of cholesterol and prenyl molecules to be employed in protein modification. We excluded the fact that in our experiments we were deleting positive modulation of 3-hydroxy-3-methylglutaryl coenzyme A (HMG-CoA) reductase, a key enzyme in the mevalonate cascade because the levels of one of the products of the pathway, ubiquinone (CoQ), were not affected by VDR silencing. Instead, we ascribed the hampered biosynthetic rate to decreased acetyl-CoA availability, and this possibility was validated by the observation that VDR silencing also abated histone acetylation. The insensitivity of ubiquinone synthesis to a decrease in acetyl-coA levels, which is critical for other lipids, may be due to differences in the Km values of the branch-point enzymes in the mevalonate pathway. The principle of the classical flow diversion hypothesis indicates that variations in the size of the precursor pool will primarily influence cholesterol synthesis because the Km of squalene synthase for its substrate is high, whereas all of the other branch-point enzymes exhibit low Km values and the rate-limiting enzymes of the CoQ biosynthetic branch may have the lowest Km values. Unfortunately, the complete details of CoQ synthesis in animal tissues remain to be elucidated.

We concluded that the VDR, by restraining mitochondrial respiratory activity, spares mitochondrial metabolic intermediates, which can be diverted from oxidative metabolism toward a biosynthetic fate. The end products that were found to be affected by this switch in the present study are essential for proliferation; in particular, cholesterol, which is continuously incorporated into membranes, and several reports indicate that cholesterogenesis is vastly elevated in various cancer cells [26]–[28]. In addition, prenylation is a post-translational modification of several small GTPases and is essential for the docking and activity of these enzymes; hampering GTPase activity interferes with proliferation, which has been extensively reported for the two small GTPases that were analyzed in the present study, RhoA and Ras, the prenylation of which is required for their ability to induce malignant transformation, invasion, and metastasis [29]. Finally, histone acetylation status is an important aspect of proliferation because it represents an epigenetic strategy controlling chromatin remodeling. Cancer cells display an impaired balance of acetylation and deacetylation reactions, which results in altered acetylation patterns and can affect gene expression [30]. Indeed, in various cancers, altered expression of histone acetyltransferases and other histone modifiers can be observed [31]. Moreover, ATP-citrate lyase, a cytosolic enzyme that catalyzes the generation of acetyl-CoA from citrate of mitochondrial origin, is upregulated in cancer and its inhibition suppresses the proliferation of various types of tumor cells (as reviewed in [32]), making this enzyme a therapeutic target for cancer. Taken together, these evidences support the fact that the acetyl-CoA that is produced in the cytosol is a crucial component of several biosynthetic pathways that promote cell growth, and our study describes the VDR as a promoter of acetyl-CoA utilization outside of the mitochondria for the first time.

Many studies have reported the reliance of cancer cells on glucose under aerobic conditions, a phenomenon known as the Warburg effect [33], [34]. In addition, another key point that is crucial for the generation of anabolic metabolites upon the tumoral metabolic switch is the diversion of TCA cycle intermediates toward biosynthetic pathways. Quiescent cells primarily utilize the TCA cycle to oxidize nutrients, generating NADH and FADH2 to fuel ATP production through the mitochondrial electron transport chain, whereas proliferating cells use the TCA cycle to provide the building blocks that are necessary to support cell growth. In this manner, mitochondrial pathways are rewired to sustain proliferation. Consequently, metabolic rearrangements that alter the balance between oxidation and the removal of metabolites for biosynthetic purposes play important roles in cancer growth.

Few reports support the idea that decreasing respiratory chain activity promotes tumor growth in cancer cells. For example, enhancement of complex I activity through NADH dehydrogenase expression strongly interferes with tumor growth and metastasis, while inhibition of complex I enhances the metastatic potential of already aggressive breast cancer cells [35]. Oncogene activity (e.g., K-Ras transformation) can decrease mitochondrial complex I activity, supporting a malignant phenotype [36]. A variety of human tumors display reduced SIRT3 expression, supporting the hypothesis that sirtuin 3 (SIRT3) acts as a tumor suppressor in humans [37]; because the deacetylase enzyme SIRT3 targets enzymes that are involved in multiple mitochondrial oxidative pathways, with the cumulative effect of promoting nutrient oxidation and energy production, its anti-oncogenic activity may reside in enhancing oxidative metabolism to the detriment of the efflux of TCA cycle metabolites for anabolic purposes.

Based on these considerations, the VDR may be regarded as a mitochondria-targeting tumor facilitator, similar to the role proposed for the leptin receptor, the signaling of which supports cancer cell metabolism by suppressing mitochondrial respiration [38].

In our experimental model, we were unable to discriminate between direct or indirect nuclear-triggered control of mitochondrial transcription by the VDR. Our observation that both nuclear- and mitochondrially encoded COX transcripts are modulated by VDR activity may be explained by concerted mitochondrial and nuclear transcriptional control that is exerted by the VDR, although we cannot exclude the possibility of nuclear signaling by the receptor, followed by cross-talk with the mitochondrial transcription machinery. However, given the abundant presence of the VDR in the mitochondrial compartment and its similar action to that reported for other steroid receptors docking at responsive elements on mtDNA, one may hypothesize that direct binding of the receptor to mtDNA occurs. Our i*n silico* analysis of VDRE sites on the mitochondrial genome has provided strong indications of direct transcriptional control that is exerted by the VDR on mtDNA. Further studies will demonstrate the accuracy of this prediction. The binding and transcriptional control remains to be demonstrated experimentally in future investigations. In our opinion, it is reasonable to conclude that nuclear and mitochondrial VDR signaling are integrated, as described for the glucocorticoid receptor [23], and that further studies will demonstrate both direct and indirect modalities of VDR action on mitochondrial transcription.

The even distribution of VDR between nucleus and mitochondria observed in HaCaT cells in our previous work [12] is justified by the general role exerted by the receptor on transcription, both at genomic and mitochondrial level. The latter has been investigated for the first time in this work. This equal nuclear and mitochondrial localization might be found in cancer cells unresponsive to differentiating actions of vitamin D and relying on mitochondrial metabolites to sustain proliferation.

In conclusion, in the present study, we discovered a strikingly important new role for the receptor in mitochondrial function and metabolism in cancer cells that are resistant to the nuclear-differentiating effects of vitamin D. Genetic silencing of the receptor produces a phenotype that differs from wild type cells in at least three major features: a decreased proliferation rate, potentiated mitochondrial respiratory chain protein levels and reduced biosynthetic capacity. These effects have been interpreted and fit well together, ascribing a novel function to the VDR in cellular metabolism. We propose that the VDR acts as a mitochondria-targeting tumor facilitator, the signaling of which supports cancer cell metabolism through the suppression of mitochondrial respiration and rewiring of metabolic intermediates toward biosynthesis.

## Methods

### Cell culture and treatment

An immortalized human epidermal keratinocyte cell line (HaCaT), the MCF7 human breast cancer cell line and the HeLa human cervical carcinoma cell line were purchased from American Type Culture Collection (ATCC), USA, and were cultured in Dulbecco's modified Eagle's medium (DMEM) that had been supplemented with 10% fetal bovine serum and 1% antibiotics [penicillin-streptomycin (Sigma-Aldrich)] at 37°C in a humidified atmosphere containing 5% CO_2_. When treated, the cells were maintained in DMEM that had been supplemented with 1% fetal bovine serum and were either incubated for the reported time with 10 nM 1,25 (OH)_2_ vitamin D3, for one hour with 10 mM H_2_O_2_, or for three hours with 0.5 M sorbitol. All of the reagents were obtained from Sigma. Fully differentiated and quiescent primary keratinocytes were obtained from Skin Bank, Ospedale CTO, Turin, Italy, and were prepared as previously reported [39]. RD cells and RD18 NpBI-206 cells were kindly provided by Prof. Carola Ponzetto. The cells were grown in DMEM that had been supplemented with 10% FBS. The RD18 NpBI-206 cells carry a doxycycline-inducible miR-206-expressing lentiviral vector [16]. For the differentiation experiments, the cells were constantly maintained in high serum-containing media (10% FBS) in the presence or absence of doxycycline (1 µg/ml) for the indicated number of days (induced miR-206, IND; uninduced miR-206, NI).

### Lentiviral-mediated shRNA targeting

PLKO.1 lentiviral shRNA clones targeting the human VDR and a scrambled non-targeting control were purchased from Sigma (Sigma Mission shRNA). The efficiency of the individual lentiviral shRNA clones in the cells was determined using real-time RT-PCR and western blotting analyses. The sequences of shRNA 3 (TRCN0000019506), shRNA 4 (TRCN0000276543), and shRNA 2 (TRCN0000019505) were as follows:

5′-CCGGCCTCCAGTTCGTGTGAATGATCTCGAGATCATTCACACGAACTGGAGGTTTTT-3′,

5′-CCGGCTCCTGCCTACTCACGATAAACTCGAGTTTATCGTGAGTAGGCAGGAGTTTTTG-3′, and

5′-CCGGGTCATCATGTTGCGCTCCAATCTCGAGATTGGAGCGCAACATGATGACTTTTT-3′.

Lentiviral transduction particles were produced in HEK293T cells by co-transfection of either the control or human VDR shRNA plasmid together with the packaging vectors pMDLg/pRRE, pRSVRev, and pMD2.VSVG. Lipofectamine 2000 (Life Technologies) was used as a transfection reagent. The supernatants were harvested 30 hours after transfection, filtered through 0.22 Am pore size filters (Corning Science Products) and used immediately for overnight transduction of the cells. Puromycin selection began 24 hours after infection. Seven days after infection, the cells were seeded for experimental assays or harvested for RNA and protein analyses.

### Extract preparation and western blotting analyses

Subcellular fractionation and western blotting analyses were conducted, as previously described [12]. The protein content of the total extracts and mitochondrial fractions was quantified using the DC protein assay (Biorad), and 50 µg of total lysates or 30 µg of the mitochondrial fractions were separated using 10% SDS-PAGE and analyzed using western blotting. The proteins were immunostained with the indicated primary antibodies for 1 h at room temperature and detection of the proteins of interest was performed using peroxidase-conjugated secondary antibodies (Pierce, Rockford, IL), followed by ECL detection (ECL detection kit, Perkin Elmer Life Science, USA). Tubulin and VDAC expression was used as loading control of total and mitochondrial extracts, respectively. In order to check the absence of cytosolic VDR contamination in mitochondrial extracts, tubulin levels were evaluated in mitochondrial fractions and were not detectable, excluding cytosolic contamination in mitochondrial preparations. An anti-VDAC (anti-porin 31HL) monoclonal antibody was purchased from Calbiochem. Mouse anti-VDR (sc-13133), anti-actin (sc-8432) and anti-tubulin (sc-53646) monoclonal antibodies, as well as rabbit anti-rhoA (sc-179) and anti-H-ras (sc-520) antibodies, were purchased from Santa Cruz, CA, USA. The rabbit anti-acetyl-histone H4 antibody (06-598) was obtained from Upstate (Millipore).

### RNA extraction and real-time PCR

RNA was extracted using TRIzol (Invitrogen) and 1 µg of total RNA that had been treated with DNase (Roche) was reverse transcribed using the iScript cDNA Synthesis Kit (Bio-Rad) according to the manufacturer's recommended protocol. Real-time PCR was performed using iQ SYBR Green (Bio-Rad) with the following primers:

VDR, fwd 5′-ACTTGTGGGGTGTGTGGAGAC-3′, rev 5′-GGCGTCGGTTGTCCTTCG-3′; COXII, fwd 5′-CGACTACGGCGGACTAATCT-3′, rev 5′-TCGATTGTCAACGTCAAGGA-3′; COXIV, fwd 5′-CGAGCAATTTCCACCTCTGT-3′, rev 5′-GGTCAGCCGATCCATATAA-3′; and β-actin, fwd 5′-CATGTACGTTGCTATCCAGGC-3′, rev 5′-CTCCTTAATGTCACGCACGAT-3′

Beta-actin was used as an internal control. The real-time PCR parameters were as follows: Cycle 1, 50°C for 2 minutes; cycle 2, 95°C for 10 minutes, followed by 45 cycles at 95°C for 15 seconds and then 60°C for 1 minute. The 2-ΔΔCT method was used to analyze the data.

### Cell proliferation assay

The cells (2000, 1000 or 500) were seeded on 96 multiwell plates and cultured for 2, 3 or 5 days. At the end of this period, the cells were fixed for 15 min with 11% glutaraldehyde and the plates were washed three times, air-dried and stained for 20 min with a 0.1% crystal violet solution. The plates were then extensively washed and air-dried prior to solubilization of the bound dye with a 10% acetic acid solution. The absorbance was determined at 595 nm. The data collected from six wells were averaged for each experimental condition.

### Flow cytometric cell cycle analyses

The cells were harvested using trypsinization, washed with PBS, and stained with 500 µl of a propidium iodide solution containing PI (80 µg/ml) and RNase A (100 µg/ml) in 3.8 mM sodium citrate. The samples were incubated in the dark for 15 min and then analyzed using a FACScan flow cytometer. The cell cycle distribution in the G0/G1, S and G2/M phases was calculated using CellQuest software (BD Pharmingen Biosciences).

### Determination of Cell Viability

Cell viability was assessed using the MTT assay [40]. This assay provides a rapid and precise method of quantifying cell viability by spectrophotometrically measuring the ability of living cells to reduce the MTT reagent. The cells were seeded in 24 well culture plates and allowed to attach for 24 hours. Then, 50 µl of MTT solution [5 mg/ml of 3-(4,5-dimethylthiazol-2-yl)-2,5-diphenyltetrazolium bromide (MTT, Sigma) in PBS] was added to each well and incubated at 37°C for 4 h. The medium was then removed, and 500 µl of DMSO was added to each well. The plates were then gently shaken for 10 min to completely dissolve the precipitates. The absorbance was detected at 570 nm (Bio-Rad, USA). The data collected from six wells were averaged for each experimental condition.

### Measurement of GSH

Glutathione was measured, as described by Rahman et al. [41], using a modified glutathione reductase–DTNB recycling assay. The cells were washed with PBS and 600 µl of 0.01 N HCl was added. After gentle scraping, the cells were frozen/thawed twice and the proteins were precipitated by adding 120 µl of 6.5% 5-sulfosalicylic acid to 480 µl of lysate. Each sample was placed on ice for 1 h and centrifuged for 15 min at 12,500×g (4°C). Total glutathione levels were measured in 20 µl of the cell lysate with the following reaction mix: Twenty microliters of stock buffer (143 mM NaH_2_PO_4_ and 63 mM EDTA, pH 7.4), 200 µl of daily reagent [10 mM 5,5′-dithiobis-2-nitrobenzoic acid (DTNB) and 2 mM NADPH in stock buffer], and 40 µl of glutathione reductase (8.5 U/ml). The oxidized glutathione (GSSG) content was obtained after derivatization of GSH with 2-vinylpyridine (2VP): 10 µl 2VP was added to 200 µl of cell lysate or culture supernatant and the mixture was shaken at room temperature for 1 h. Glutathione levels were then measured in 40 µl of sample, as described. The kinetics of the reaction were followed for 5 min using a Packard microplate reader EL340, measuring the absorbance at 415 nm. Each measurement was performed in triplicate. For each sample, GSH levels were obtained by subtracting the amount of GSSG from total glutathione levels.

### Measurement of the mitochondrial membrane potential (ΔΨm)

JC-1, a mitochondrial dye that stains the mitochondria in living cells in a membrane potential-dependent fashion, was used to determine ΔΨm. JC-1 is a cationic dye that indicates mitochondrial polarization by shifting its fluorescence emission from green (530 nm) to red (590 nm). The cells were harvested by trypsinization, washed with PBS and incubated with JC-1 (2 µg/ml final concentration) at 37°C for 30 minutes. After washing, JC-1 accumulation was determined using flow cytometric analysis. The amount of JC-1 retained by 10,000 cells per sample was measured at 530 nm (FL-1 green fluorescence) and 590 nm (FL-2 red fluorescence) using a flow cytometer and analyzed using Cell Quest Alias software. The ratio of FL2/FL1 was evaluated to determine ΔΨm.

### Measurement of *de novo* cholesterol and ubiquinone synthesis

The *de novo* synthesis of cholesterol and ubiquinone was measured by radiolabeling cells with 1 µCi/ml of [^3^H]acetate (3600 mCi/mmol; Amersham GE Healthcare, Piscataway, NJ), as previously reported [42]. Briefly, the cells were harvested in PBS and cellular lipids were extracted using methanol and hexane. The cellular lipid extracts that were produced by this separation were re-suspended in 30 µl of chloroform and then subjected to thin layer chromatography (TLC) using a 1∶1 (v/v) ether/hexane solution as the mobile phase. Each sample was spotted on pre-coated LK6D Whatman silica gels (Merck, Darmstadt, Germany) and allowed to run for 30 min. Solutions of 1 mg/ml of cholesterol and ubiquinone were used as standards. The silica gel plates were exposed for 1 h to an iodine-saturated atmosphere and the migrated spots were cut out. Their radioactivity was measured via liquid scintillation using a Tri-Carb Liquid Scintillation Analyzer (PerkinElmer, Waltham, MA). Cholesterol and ubiquinone synthesis were expressed as fmol of [^3^H]cholesterol or pmol of [^3^H]ubiquinone/mg of protein, according to previously prepared calibration curves.

### Assessment of small GTPase prenylation

Cells were lysed with 2% ice cold Triton X-114 in Tris buffered saline, pH 7.4, and phase-separated, as previously described [43] (with some modifications). The cells were harvested in lysis buffer (25 mM Tris-HCl, pH 7.4, 150 mM NaCl, 2% Triton X-114, 5 mM MgCl_2_, 1 mM Na_2_HPO_4_, 1 mM sodium orthovanadate, 1 mM PMSF, and protease inhibitor cocktail set III; Calbiochem) and incubated for 30 min at 4°C. After sonication, the insoluble material was removed by centrifugation at 13,000×*g* for 10 min at 4°C and the supernatants were phase-separated. Briefly, each sample was overlaid on a sucrose cushion and after warming at 37°C for 3 min, the turbid solution was centrifuged at 300×*g* for 5 min at room temperature to separate the hydrophobic and aqueous phases. Both phases were collected and the separation was repeated. The protein content of the total lysate and detergent phase were determined using the Bradford test (Bio-Rad) and aliquots were analyzed using SDS-PAGE. Anti-RhoA and anti-Ras antibodies were used to evaluate RhoA and Ras levels in the detergent phase (hydrophobic prenylated forms), whereas an anti-VDAC antibody was used to verify the partitioning of hydrophobic proteins in the detergent phase. Actin was evaluated as a loading control in the total lysates.

### Bioinformatic analysis

The Positional Weight Matrices (PWMs) were based on the consensus sequences reported in [44]. VDRE sites are formed by two core hexameric binding motifs: RGKTSA (where R = A or G, K = G or T, and S = C or G). These motifs are found with three or more intervening nucleotides repeated in a direct RGKTSA-(N)n-RGKTSA and everted RGKTSA-(N)m-ASTKGR fashion (n = 3 or 4; m = 6, 7, 8, or 9). The matrices can be found in S1 Table. Human mtDNA was downloaded from the University of California at Santa Cruz (UCSC) Genome Browser [45]. The scores were computed using an affinity approach, as described in [46], and are reported as the percentage relative to the maximum score. Affinity tests were performed with 80% (high affinity) and 60% (low affinity) cutoffs, and VDRE sites with overlapping sequences were merged to obtain unambiguous binding sites. An enrichment test was performed using Fisher's Exact Test. Hypervariable Region 1 was defined as in [47].

### Statistical analyses

The data are presented as the means ±S.D. Statistical analysis of the data was performed using either an unpaired, 2-tailed Student's t-test (for two groups) or a one-way ANOVA test with Tukey's post-hoc correction (for more than two groups). P<0.05 was considered to be significant.

## Supporting Information

S1 Fig **Vitamin D treatment does not affect HaCaT cell proliferation.** The cells were grown for 5 days in the presence or absence (control) of different concentrations of vitamin D. At the indicated times, the cells were stained with crystal violet and proliferation was quantified as the percentage of the control at the same time point. The data represent the means ±SD of three independent experiments.
(TIF)

S2 Fig **Silencing efficacy and effects of the different VDR-targeting shRNAs on proliferation.** Along with shRNA 3, which was used for all of the experiments, two additional shRNAs were tested: shRNAs 2 and 4. (**A**) The silencing efficiency of the different lentiviral shRNA clones was determined using western blot analysis of VDR expression in HaCaT cells, and tubulin expression demonstrated equivalent protein loading. (**B**) A time course proliferation assay was conducted in HaCaT cells that had been infected with the shRNA control and the three different clones. Cell growth was restrained only when the shRNA particles efficiently abated VDR expression (shRNAs 3 and 4), whereas when shRNA 2 was used, its lack of efficacy was evident both in silencing and growth inhibition. The results are displayed as the means ±SD of three independent experiments. *p<0.05 compared to the control.
(TIF)

S3 Fig **Effects of VDR silencing on intracellular GSH levels.** HaCaT cells were infected with either the shRNA control or VDR shRNA 3 and intracellular glutathione was measured seven days post-infection. The results are presented as the means ±SD of three independent experiments. *p<0.05 compared to the control.
(TIF)

S1 Table **mtDNA sequences matching VDRE site matrices in the affinity analysis.** (**A**) The complete list of the mtDNA sequences that were detected in the *in silico* analysis. Overlapping sequences were merged and are shown as one sequence with more than one predicted VDRE site. For each sequence, the matrix (representing one of the possible VDRE sites) matching the sequence, the start site of the sequence (as referred to in the UCSC database in the Methods section), the affinity score (which is shown as a percentage of the maximum score for each matrix) and the strand of the sequence are shown. For overlapping sequences, more than one matrix, start site, score and strand are reported. For VDRE sites located on the reverse strand, the sequence reported in the Table is that of the reverse strand. (**B**) The matrices used in the affinity analysis. The matrices represent all of the VDRE sites described in [44].
(DOCX)

## References

[1] Doig CL, Singh PK, Dhiman VK, Thorne JL, Battaglia S, et al (2013) Recruitment of NCOR1 to VDR target genes is enhanced in prostate cancer cells and associates with altered DNA methylation patterns. Carcinogenesis 34:248–256. 23087083 10.1093/carcin/bgs331 PMC3564435

[2] Khanim FL, Gommersall LM, Wood VH, Smith KL, Montalvo L, et al (2004) Altered SMRT levels disrupt vitamin D3 receptor signalling in prostate cancer cells. Oncogene 23:6712–6725. 15300237 10.1038/sj.onc.1207772

[3] Banwell CM, MacCartney DP, Guy M, Miles AE, Uskokovic MR, et al (2006) Altered nuclear receptor corepressor expression attenuates vitamin D receptor signaling in breast cancer cells. Clin Cancer Res 12:2004–2013. 16609009 10.1158/1078-0432.CCR-05-1218

[4] Abedin SA, Thorne JL, Battaglia S, Maguire O, Hornung LB, et al (2009) Elevated NCOR1 disrupts a network of dietary sensing nuclear receptors in bladder cancer cells. Carcinogenesis 30:449–456. 19126649 10.1093/carcin/bgp005 PMC2722152

[5] Huhtakangas J, Olivera CJ, Bishop JE, Zanello LP, Norman AW (2004) The vitamin D receptor is present in caveolae-enriched plasma membranes and binds 1a,25(OH)2 vitamin D3 in vivo and in vitro. Mol Endocrinol 18:2660–2671. 15272054 10.1210/me.2004-0116

[6] Ordóñez-Morán P, Larriba MJ, Pálmer HG, Valero RA, Barbáchano A, et al (2008) RhoA-ROCK and p38MAPK-MSK1 mediate vitamin D effects on gene expression, phenotype, and Wnt pathway in colon cancer cells. J Cell Biol 183:697–710. 19015318 10.1083/jcb.200803020 PMC2582889

[7] Sequeira VB, Rybchyn MS, Tongkao-On W, Gordon-Thomson C, Malloy PJ, et al (2012) The role of the vitamin D receptor and ERp57 in photoprotection by 1α,25-dihydroxyvitamin D3. Mol Endocrinol 26:574–582. 22322599 10.1210/me.2011-1161 PMC3327356

[8] Gavrilova-Jordan LP, Price TM (2007) Actions of Steroids in Mitochondria. Semin Reprod Med 25:154–164. 17447205 10.1055/s-2007-973428

[9] Morrish F, Buroker NE, Ge M, Ning XH, Lopez-Guisa J, et al (2006) Thyroid hormone receptor isoforms localize to cardiac mitochondrial matrix with potential for binding to receptor elements on mtDNA. Mitochondrion 6:143–148. 16730242 10.1016/j.mito.2006.04.002

[10] Chen JQ, Delannoy M, Cooke C, Yager JD (2004) Mitochondrial localization of ERα and ERβ in human MCF7 cells. Am J Physiol Endocrinol Metab 286:1011–1022. 10.1152/ajpendo.00508.2003 14736707

[11] Lee J, Sharma S, Kim J, Ferrante RJ, Ryu H (2008) Mitochondrial Nuclear Receptors and Transcription Factors: Who's Minding the Cell? J Neurosci Res 86:961–971. 18041090 10.1002/jnr.21564 PMC2670446

[12] Silvagno F, Consiglio M, Foglizzo V, Destefanis M, Pescarmona G (2013) Mitochondrial translocation of vitamin D receptor is mediated by the permeability transition pore in human keratinocyte cell line. PLoS One 8:e54716. 23349955 10.1371/journal.pone.0054716 PMC3551909

[13] Galluzzi L, Morselli E, Kepp O, Vitale I, Rigoni A, et al (2010) Mitochondrial gateways to cancer. Mol Aspects Med 31:1–20. 19698742 10.1016/j.mam.2009.08.002

[14] Hanahan D, Weinberg RA (2011) Hallmarks of cancer: the next generation Cell. 144:646–674. 10.1016/j.cell.2011.02.013 21376230

[15] Schulze A, Harris AL (2012) How cancer metabolism is tuned for proliferation and vulnerable to disruption. Nature 491:364–373. 23151579 10.1038/nature11706

[16] Taulli R, Bersani F, Foglizzo V, Linari A, Vigna E, et al (2009) The muscle-specific microRNA miR-206 blocks human rhabdomyosarcoma growth in xenotransplanted mice by promoting myogenic differentiation. J Clin Invest 119:2366–2378. 19620785 10.1172/JCI38075 PMC2719932

[17] Lee SR, Kim HK, Song IS, Youm J, Dizon LA, et al (2013) Glucocorticoids and their receptors: insights into specific roles in mitochondria. Prog Biophys Mol Biol 112:44–54. 23603102 10.1016/j.pbiomolbio.2013.04.001

[18] Psarra AMG, Sekeris CE (2009) Glucocorticoid receptors and other nuclear transcription factors in mitochondria and possible functions. Biochim Biophys Acta 1787:431–436. 19100710 10.1016/j.bbabio.2008.11.011

[19] Chen JQ, Cammarata PR, Baines CP, Yager JD (2009) Regulation of mitochondrial respiratory chain biogenesis by estrogens/estrogen receptors and physiological, pathological and pharmacological implications. Biochim Biophys Acta 1793:1540–1570. 19559056 10.1016/j.bbamcr.2009.06.001 PMC2744640

[20] Ryan MT, Hoogenraad NJ (2007) Mitochondrial-nuclear communications. Annu Rev Biochem 76:701–722. 17227225 10.1146/annurev.biochem.76.052305.091720

[21] Cioffi F, Senese R, Lanni A, Goglia F (2013) Thyroid hormones and mitochondria: with a brief look at derivatives and analogues. Mol Cell Endocrinol 379:51–61. 23769708 10.1016/j.mce.2013.06.006

[22] Vasconsuelo A, Milanesi L, Boland R (2013) Actions of 17β-estradiol and testosterone in the mitochondria and their implications in aging. Ageing Res Rev 12:907–917. 24041489 10.1016/j.arr.2013.09.001

[23] Psarra AMG, Sekeris CE (2011) Glucocorticoids induce mitochondrial gene transcription in HepG2 cells: role of the mitochondrial glucocorticoid receptor. Biochim Biophys Acta 1813:1814–1821. 21664385 10.1016/j.bbamcr.2011.05.014

[24] Abedin SA, Banwell CM, Colston KW, Carlberg C, Campbell MJ (2006) Epigenetic corruption of VDR signalling in malignancy. Anticancer Res 26:2557–2566. 16886664

[25] Bikle DD, Oda Y, Xie Z (2005) Vitamin D and skin cancer: a problem in gene regulation. J Steroid Biochem Mol Biol 97:83–91. 16039846 10.1016/j.jsbmb.2005.06.001

[26] Mares-Perlman JA, Shrago E (1988) Energy substrate utilization in freshly isolated Morris Hepatoma 7777 cells. Cancer Res 48:602–608. 3335023

[27] Murtola TJ, Syvälä H, Pennanen P, Bläuer M, Solakivi T, et al (2012) The importance of LDL and cholesterol metabolism for prostate epithelial cell growth. PLoS One 7:e39445. 22761797 10.1371/journal.pone.0039445 PMC3384647

[28] Campbell AM, Chan SH (2008) Mitochondrial membrane cholesterol, the voltage dependent anion channel (VDAC), and the Warburg effect. J Bioenerg Biomembr 40:193–197. 18677555 10.1007/s10863-008-9138-x

[29] Berndt N, Hamilton AD, Sebti SM (2011) Targeting protein prenylation for cancer therapy. Nat Rev Cancer 11:775–791. 22020205 10.1038/nrc3151 PMC4037130

[30] Di Cerbo V, Schneider R (2013) Cancers with wrong HATs: the impact of acetylation. Brief Funct Genomics 12:231–243. 23325510 10.1093/bfgp/els065

[31] Ozdağ H, Teschendorff AE, Ahmed AA, Hyland SJ, Blenkiron C, et al (2006) Differential expression of selected histone modifier genes in human solid cancers. BMC Genomics 7:90. 16638127 10.1186/1471-2164-7-90 PMC1475574

[32] Zaidi N, Swinnen JV, Smans K (2012) ATP-citrate lyase: a key player in cancer metabolism. Cancer Res 72:3709–3714. 22787121 10.1158/0008-5472.CAN-11-4112

[33] Jones RG, Thompson CB (2009) Tumor suppressors and cell metabolism: a recipe for cancer growth. Genes Dev 23:537–548. 19270154 10.1101/gad.1756509 PMC2763495

[34] Vander Heiden MG, Cantley LC, Thompson CB (2009) Understanding the Warburg effect: the metabolic requirements of cell proliferation. Science 324:1029–1033. 19460998 10.1126/science.1160809 PMC2849637

[35] Santidrian AF, Matsuno-Yagi A, Ritland M, Seo BB, LeBoeuf SE, et al (2013) Mitochondrial complex I activity and NAD+/NADH balance regulate breast cancer progression. J Clin Invest 123:1068–1081. 23426180 10.1172/JCI64264 PMC3582128

[36] Hu Y, Lu W, Chen G, Wang P, Chen Z, et al (2012) K-ras (G12V) transformation leads to mitochondrial dysfunction and a metabolic switch from oxidative phosphorylation to glycolysis. Cell Res 22:399–412. 21876558 10.1038/cr.2011.145 PMC3257361

[37] Kim HS, Patel K, Muldoon-Jacobs K, Bisht KS, Aykin-Burns N, et al (2010) SIRT3 is a mitochondria-localized tumor suppressor required for maintenance of mitochondrial integrity and metabolism during stress. Cancer Cell 17:41–52. 20129246 10.1016/j.ccr.2009.11.023 PMC3711519

[38] Park J, Kusminski CM, Chua SC, Scherer PE (2010) Leptin receptor signaling supports cancer cell metabolism through suppression of mitochondrial respiration in vivo. Am J Pathol 177:3133–3144. 21056997 10.2353/ajpath.2010.100595 PMC2993284

[39] Castagnoli C, Fumagalli M, Alotto D, Cambieri I, Casarin S, et al**.** (2010) Preparation and characterization of a novel skin substitute. J Biomed Biotechnol 2010. pii: 840363. 10.1155/2010/840363 PMC2946634 20936183

[40] Mosmann T (1983) Rapid colorimetric assay for cellular growth and survival: application to proliferation and cytotoxicity assays. J Immunol Methods 65:55–63. 6606682 10.1016/0022-1759(83)90303-4

[41] Rahman I, Kode A, Biswas SK (2006) Assay for quantitative determination of glutathione and glutathione disulfide levels using enzymatic recycling method. Nat Protoc 1:3159–3165. 17406579 10.1038/nprot.2006.378

[42] Campia I, Gazzano E, Pescarmona G, Ghigo D, Bosia A. et al. (2009) Digoxin and ouabain increase the synthesis of cholesterol in human liver cells. Cell Mol Life Sci 66:1580–1594. 19288057 10.1007/s00018-009-9018-5 PMC11131534

[43] Bordier C (1981) Phase separation of integral membrane proteins in Triton X-114 solution. J Biol Chem 256:1604–1607. 6257680

[44] Carlberg C, Seuter S (2007) The Vitamin D Receptor. Dermatologic Clinics 25:515–523. 17903610 10.1016/j.det.2007.06.004

[45] Karolchik D, Barber GP, Casper J, Clawson H, Cline MS, et al (2014) The UCSC Genome Browser database: 2014 update. Nucleic Acids Res 42:764–770.24157835

[46] Wasserman WW, Sandelin A (2004) Applied bioinformatics for the identification of regulatory elements. Nature 5:276–287. 10.1038/nrg1315 15131651

[47] Stoneking M (2000) Hypervariable sites in the mtDNA control region are mutational hotspots. Am J Hum Genet 67:1029–1032. 10968778 10.1086/303092 PMC1287875

