# Supplementary figures and images for: The Vitamin D Receptor Inhibits the Respiratory Chain, Contributing to the Metabolic Switch that Is Essential for Cancer Cell Proliferation
Source: PLoS One. 2014 Dec 29;9(12):e115816. doi: 10.1371/journal.pone.0115816 (PMC4278832; doi:10.1371/journal.pone.0115816)

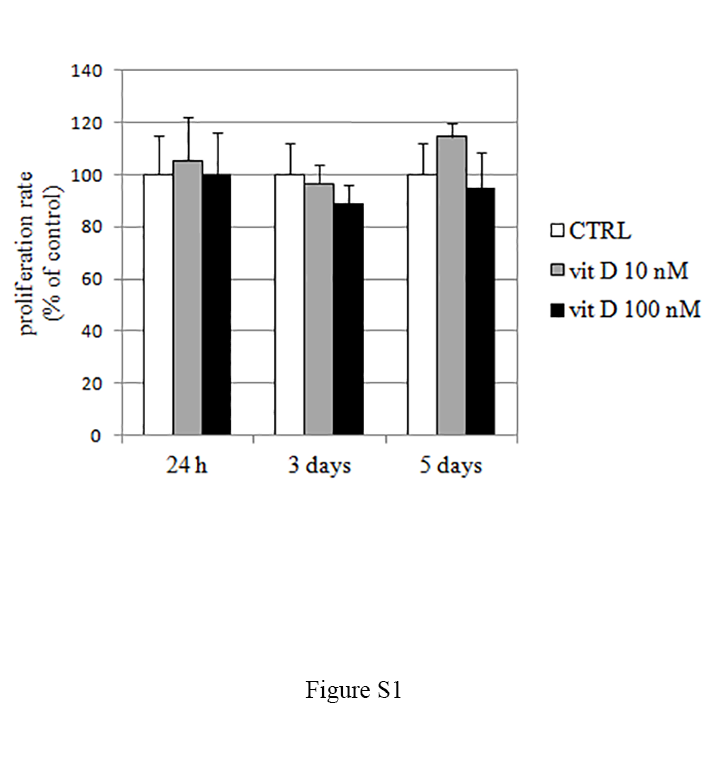

Supplement: S1 Fig — Vitamin D treatment does not affect HaCaT cell proliferation. The cells were grown for 5 days in the presence or absence (control) of different concentrations of vitamin D. At the indicated times, the cells were stained with crystal violet and proliferation was quantified as the percentage of the control at the same time point. The data represent the means ±SD of three independent experiments. (TIF) [file pone.0115816.s001.tif]

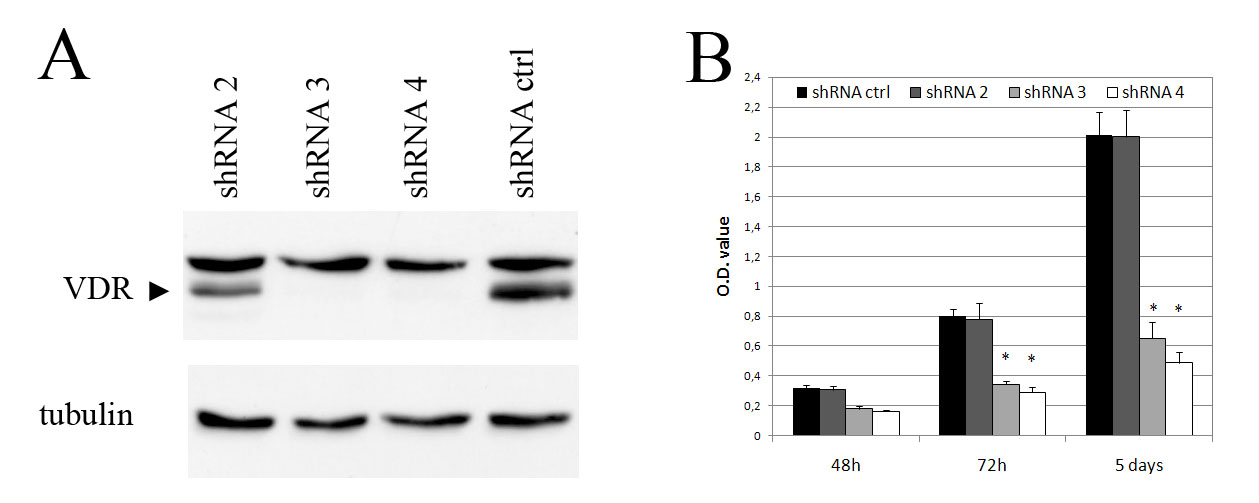

Supplement: S2 Fig — Silencing efficacy and effects of the different VDR-targeting shRNAs on proliferation. Along with shRNA 3, which was used for all of the experiments, two additional shRNAs were tested: shRNAs 2 and 4. (A) The silencing efficiency of the different lentiviral shRNA clones was determined using western blot analysis of VDR expression in HaCaT cells, and tubulin expression demonstrated equivalent protein loading. (B) A time course proliferation assay was conducted in HaCaT cells that had been infected with the shRNA control and the three different clones. Cell growth was restrained only when the shRNA particles efficiently abated VDR expression (shRNAs 3 and 4), whereas when shRNA 2 was used, its lack of efficacy was evident both in silencing and growth inhibition. The results are displayed as the means ±SD of three independent experiments. *p<0.05 compared to the control. (TIF) [file pone.0115816.s002.tif]

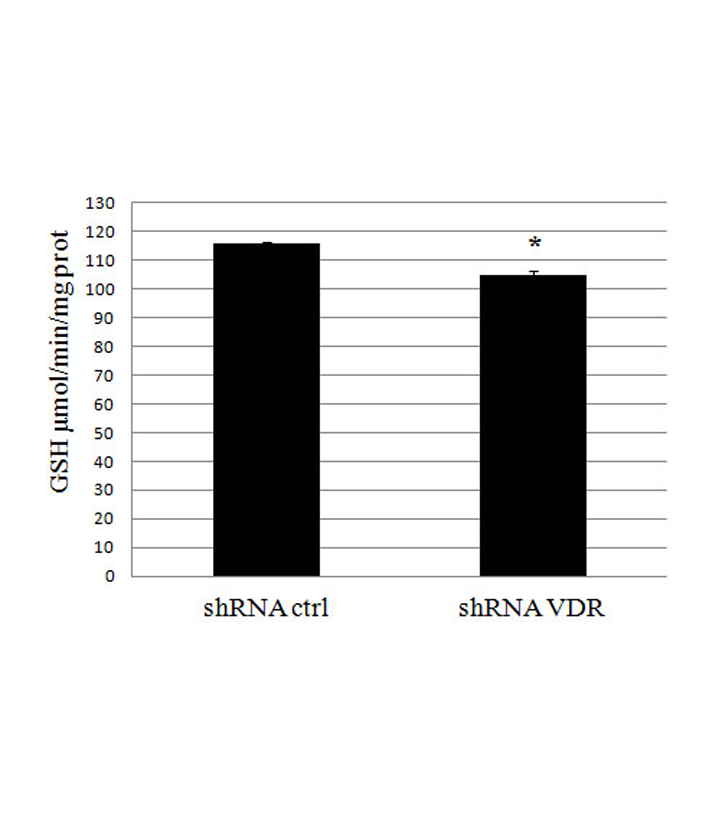

Supplement: S3 Fig — Effects of VDR silencing on intracellular GSH levels. HaCaT cells were infected with either the shRNA control or VDR shRNA 3 and intracellular glutathione was measured seven days post-infection. The results are presented as the means ±SD of three independent experiments. *p<0.05 compared to the control. (TIF) [file pone.0115816.s003.tif]
