# Supplementary material for: The Vitamin D Receptor Inhibits the Respiratory Chain, Contributing to the Metabolic Switch that Is Essential for Cancer Cell Proliferation
Source: PLoS One. 2014 Dec 29;9(12):e115816. doi: 10.1371/journal.pone.0115816 (PMC4278832; doi:10.1371/journal.pone.0115816)
Supplement: S1 Table — mtDNA sequences matching VDRE site matrices in the affinity analysis. (A) The complete list of the mtDNA sequences that were detected in the in silico analysis. Overlapping sequences were merged and are shown as one sequence with more than one predicted VDRE site. For each sequence, the matrix (representing one of the possible VDRE sites) matching the sequence, the start site of the sequence (as referred to in the UCSC database in the Methods section), the affinity score (which is shown as a percentage of the maximum score for each matrix) and the strand of the sequence are shown. For overlapping sequences, more than one matrix, start site, score and strand are reported. For VDRE sites located on the reverse strand, the sequence reported in the Table is that of the reverse strand. (B) The matrices used in the affinity analysis. The matrices represent all of the VDRE sites described in [44]. (DOCX) [file pone.0115816.s004.docx]

**A**

**B**

| DR3 | RGKTSANNNRGKTSA |
| --- | --- |
| DR4 | RGKTSANNNNRGKTSA |
| EV6 | RGKTSANNNNNNASTKGR |
| EV7 | RGKTSANNNNNNNASTKGR |
| EV8 | RGKTSANNNNNNNNASTKGR |
| EV9 | RGKTSANNNNNNNNNASTKGR |

(R = A or G, K = G or T, and S = C or G)

Supplementary Table 1
